# Emerging Advances in Microfluidic Hydrogel Droplets for Tissue Engineering and STEM Cell Mechanobiology

**DOI:** 10.3390/gels9100790

**Published:** 2023-10-01

**Authors:** Mohamad Orabi, Joe F. Lo

**Affiliations:** Department of Mechanical Engineering, University of Michigan, 4901 Evergreen Road, Dearborn, MI 48128, USA; orabimo@umich.edu

**Keywords:** microfluidics, microchannels, droplet generation, droplet size, hydrogels

## Abstract

Hydrogel droplets are biodegradable and biocompatible materials with promising applications in tissue engineering, cell encapsulation, and clinical treatments. They represent a well-controlled microstructure to bridge the spatial divide between two-dimensional cell cultures and three-dimensional tissues, toward the recreation of entire organs. The applications of hydrogel droplets in regenerative medicine require a thorough understanding of microfluidic techniques, the biocompatibility of hydrogel materials, and droplet production and manipulation mechanisms. Although hydrogel droplets were well studied, several emerging advances promise to extend current applications to tissue engineering and beyond. Hydrogel droplets can be designed with high surface-to-volume ratios and a variety of matrix microstructures. Microfluidics provides precise control of the flow patterns required for droplet generation, leading to tight distributions of particle size, shape, matrix, and mechanical properties in the resultant microparticles. This review focuses on recent advances in microfluidic hydrogel droplet generation. First, the theoretical principles of microfluidics, materials used in fabrication, and new 3D fabrication techniques were discussed. Then, the hydrogels used in droplet generation and their cell and tissue engineering applications were reviewed. Finally, droplet generation mechanisms were addressed, such as droplet production, droplet manipulation, and surfactants used to prevent coalescence. Lastly, we propose that microfluidic hydrogel droplets can enable novel shear-related tissue engineering and regeneration studies.

## 1. Introduction

### 1.1. The Promise of 3D Tissue Regeneration

The loss or failure of an organ or tissue has severe consequences for millions of people worldwide. The transplant waiting list in the United States grows by one person every 10 min, while sadly, 8000 people perish from the list each year. This means an average of 22 people per day and almost one person each hour passes because of the shortage of organs [1]. Massive investment has been applied in exploring and developing an advanced approach for repairing, regenerating, or improving dysfunctional organs or tissues [2,3]. Tissue engineering is considered one of the most advanced approaches as it uses a combination of cells, biomaterials, and relevant biomolecular and physiochemical factors to maintain or even regenerate an organ [4]. Throughout the last decade, the field of tissue engineering has developed many advanced approaches based on micro- and nanofabrication techniques [5]. Creating viable skin, bone, cartilage, bladders, and blood vessels is the most attractive topic in current biomedical research [6]. The ultimate goal of tissue engineering is to build large pieces of tissue to recreate entire organs to close the gap in the transplant shortage [5]. However, creating organs and/or tissues is challenging and requires a deep understanding of the underlying cell and biology, as well as the ability to manipulate numerous biomolecular factors in the cells [5]. One of these challenges is to create a vascular system in a 3D tissue structure to provide a steady flow of oxygen and nutrients for growth [5]. Moreover, the type of biomaterials in tissue engineering affects biodegradability and biocompatibility and is key in providing the proper environment for cell growth in 3D structures. These biomaterials can imitate many features of the extracellular matrix (ECM) found in tissues, thereby controlling cell behavior and facilitating the functional restoration of damaged tissues [7,8]. Shearing and biomechanical cues improve angiogenesis and cell differentiation in tissue engineering by mimicking the physical microenvironment of native tissue and promoting the formation of differentiated cells [9]. Shearing forces, generated by fluid flow or mechanical stress, stimulate cell alignment and remodeling, leading to improved cell viability and function [10]. Biomechanical cues also regulate cell behavior and promote tissue maturation [9]. By incorporating these cues in tissue engineering approaches, researchers can improve the formation of functional tissues and enhance tissue repair and regeneration.

### 1.2. Employing Hydrogel Droplets in Tissue Engineering

The research effort to employ hydrogels and biomaterials in tissue engineering and biomedicine started in the early 1960s when Wichterle et al. proposed the application of a hydrated hydroxymethyl methacrylate (HEMA) network to contact lenses [11,12]. By that time, hydrogels known as hydrophilic materials maintained their physiochemical properties and mimicked many characteristics of the ECM found in tissues [13,14,15,16]. Furthermore, the gelation of hydrogels is very flexible and tunable where the crosslinking happens through different techniques such as physical, dynamic, and chemical crosslinking, which leveraged cell encapsulation, droplet formation, and bioprinting applications [17,18]. A deep understanding of the molecular level of hydrogels gives us detailed characteristics and functionalities including tunable properties, excellent biocompatibility, controllable degradability, and mechanical matching with biological issues [19,20]. The attractive features of hydrogels make them an exceptional bridging material to regenerative medicine, where scaffolds, droplets, and hydrogel resins are used to create 3D tissue constructs [21,22]. Conventional droplet generators are used widely to produce small droplets under controlled conditions. Some common types of these generators are pressure-driven droplet generators in which they use a pressure source to break a continuous stream of liquid into droplets and can be further classified into devices such as nozzles, capillaries, and needle-based systems [23]. Despite the wide usage of pressure droplet generators, their limitations in terms of droplet size control and handling of viscous liquids led to the development of other improved control and versatility generators [23]. Electrospray droplet generators rely on the application of an electric field to generate droplets from a liquid in a continuous stream; however, their sensitivity to the properties of the liquid being processed and the need for specialized equipment limits their practicality and versatility in some biomedical applications [24]. Pneumatic generators use compressed air to atomize a liquid stream into droplets while sonication droplet generators use ultrasound to break a liquid into droplets [25,26]. They share the major drawbacks of pressure droplet generators in controlling droplet size and handling viscous liquids [23,25,26].

### 1.3. Studying the Interfacial Adhesivity of Hydrogels

**Figure 1:**
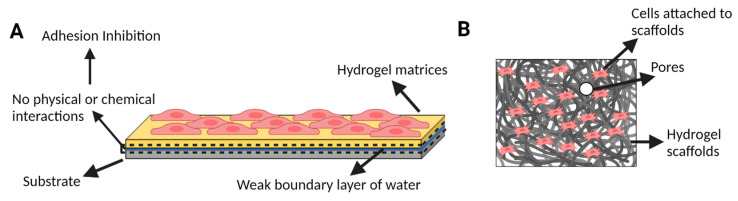
Illustration of (**A**) the weak boundary layers made of water that inhibit the direct surface between hydrogels and substrates. The presence of a significant amount of water in hydrogel matrices creates a thin, weak boundary layer that hinders direct surface contact between the hydrogels and substrates. Consequently, this leads to a reduction in surface energy and adhesive strength. (**B**) The attachment and adhesion of cells with porous scaffolds. The surfaces and interfaces of biomaterials/hydrogels directly engage with cells and tissues and exert a substantial impact on various cellular behaviors, including adhesion, spreading, proliferation, migration, and differentiation.

In the aforementioned tissue engineering, droplet generation and bioprinting applications require hydrogels to interact with the different material surfaces. The abundance of water in hydrogel matrices produces a weak boundary layer that inhibits the direct surface contact between hydrogels and substrates and, thus, results in diminished surface energy and adhesion strength (Figure 1A) [27,28]. Therefore, conventional hydrogels have limited adhesive strength toward wet and dynamic biological tissues. Tissue environments are known as complex and multitudinous, which deteriorates further the strong adhesion strength of hydrogels [29,30]. Different unique strategies are developed to overcome these challenges and manipulate molecular-scale interactions within hydrogel networks to produce a preferable interfacial adhesion in dynamic and turbulent environments. Zhang et al. investigated the mussel-inspired chemistry for the rational design of adhesive hydrogels [31]. However, to study the adhesion between hydrogels and diverse nonporous surfaces, Yuk et al. generated a set of powerful fabrication methods that have extremely tough and functional adhesion [32]. The use of supramolecular interactions to construct bioinspired underwater adhesives is discussed by Hofman et al. [28]. Therefore, for most tissue engineering and regenerative medicine applications, the tough interfacial adhesion between hydrogels and tissues is one of the most important factors that promise the overall robustness and reliability of hydrogels [31,33,34,35]. Duan et al. leveraged a multilayered microfluidic approach to integrate a novel oxygen gradient (0–20%) with an enhanced hydrogel sensor to study pancreatic beta cells [36]. A hydrogel–PDMS adhesion is used to fabricate the device and to achieve spatiotemporal detection. Evidence supporting the current islet oscillator model is reported, in addition to presenting a new relationship between oxygen and beta cell oscillations with an optimum oxygen level between 10 and 12% [36].

### 1.4. Microfluidic Control of Hydrogel Properties Promotes Cell Growth and Differentiation

The surfaces and interfaces of biomaterials/hydrogels interact directly with cells and tissues (Figure 1B) and significantly influence cellular behaviors such as adhesion, spreading, proliferation, migration, and differentiation, in addition to the outcomes of tissue repair [37]. To provide the required mechanical and chemical signals, cells are seeded in a scaffold constructed from biomaterials designed to regulate cell adhesion, proliferation, and differentiation and are assembled into three-dimensional tissues [38]. Porous scaffolds with three-dimensional microstructures are considered for different tissue engineering applications, especially cartilage tissue engineering. However, preparing porous scaffolds with uniform three-dimensional holes and high interconnectivity is difficult [37]. Different techniques are followed for the preparation of porous scaffolds such as bioprinting [39] and microfluidic procedures [40,41]. Recent studies have used conventional microfluidic techniques designed and employed to fabricate scaffolds efficiently with a desirable homogenous porosity, uniform spatial structure, interconnectivity, and potential mechanical properties [40,41]. New materials that encapsulate target cells in the small spherical porous foam are fabricated using these microfluidic techniques, representing a novel, powerful method for tissue engineering [40,41]. The mechanical stiffness of hydrogels can significantly impact the differentiation of cells. MSCs, for example, are influenced directly by the physical microenvironment of hydrogels and can differentiate into various cell types [42]. Hydrogels with high mechanical stiffness can promote the differentiation of MSCs toward a more rigid tissue such as bone [43], while hydrogels with low mechanical stiffness can promote the differentiation toward more flexible tissue like cartilage [42,43]. Studies have shown that MSCs exposed to stiff hydrogels exhibit increased expression of osteogenic markers and mineralization [44], while MSCs exposed to soft hydrogels exhibit increased expression of chondrogenic markers and cartilage formation [42]. Therefore, the mechanical stiffness of hydrogels can be utilized as a tool to control MSC differentiation and promote the formation of specific tissues for tissue engineering applications [42].

Microfluidics is considered a unique technique to prepare hydrogel microparticles because of the ease of controlling the size and shape of microparticles [45]. Using double emulsion techniques or photolithography, while combining different streams, composite particles can be manufactured with high precision in control reaction and heat and mass transport rates and control of the characteristics of the microparticles [46,47]. For tissue engineering and regenerative medicine applications, particle fabrication can be integrated with the sensors, heaters, coolers, and elements needed for biological experiments [48]. Shao et al. [49] conducted an extensive analysis of the production of microcarriers utilizing droplet microfluidics and explored their utilization in the realm of biomedicine. Their comprehensive examination primarily centered on the production of biomedical microcarriers derived from single-, double-, and multiple-emulsion droplets. Concurrently, Zheng et al. [50] conducted a review encompassing the current advancements in microfluidic-based droplet techniques for the synthesis of functional materials. Their review provided insight into the diverse applications of these techniques in the manipulation of cell fate and function. In contrast, our review provides a comprehensive overview of various microfluidic fabrication techniques in conjunction with the utilization of biomaterials and hydrogels for the creation of hydrogel microparticles. Additionally, we delve into the intricate interplay between hydrogels and microfluidic devices, while also addressing the ramifications of shear stress on cell physiology.

## 2. Short Treatise on Microfluidics Principles

Here, we provide a short introduction to microfluidics in the context of hydrogel droplet generation. Microfluidics refers to both the fundamental physics and applied technologies to manipulate fluids at the micron scale. It is generated following the engineering of the device and the physics of fluids’ principles with very small footprints and microchannels. The concept behind microfluidics is to implement operations in a single micro-sized device, encouraging scientists to pursue the challenge of microelectronics. As microelectronics is the science and the engineering of electronic components below the micron scale, microfluidics is essentially a field dedicated to miniaturized flows and fluidic manipulation [48]. However, the fundamental physics behind microfluidics changes more rapidly with the size scale of the system, which is not the case with microelectronics. At the nanoscale, quantum mechanical effects become more prominent and lead to changes in the behavior of electrons compared to classical physics. In contrast, fluids inside microchannels of microfluidic devices gain new features at this scale. One of these features is the laminar flow regime, where viscosity-related effects are more important than inertial ones [48]. The characteristic of this regime is that the fluids mix primarily via diffusion, a time-limited mechanism that impedes biochemical reactions. However, this laminar regime creates a predictable flow pattern that keeps particle generation and trajectory stable throughout the microfluidic device. In turn, the predictability of reagent flow creates a uniform distribution of size and volume for the generated hydrogel droplets that can be used in tissue engineering.

Within a microfluidic device, several phenomena can happen simultaneously, which complicates the physics of fluids inside these devices. To express which one of these phenomena is dominating, Reynolds number “Re” is used, which is given by the ratio of inertial forces over vicious ones [51]. The bodies in microfluidic devices are continuum materials, and to better describe the motion of fluids in these devices, properties like mass and force must be replaced with their continuum counterparts such as density (δ) and force (f) per unit volume [50]. The forces acting on each fluid element are due to fluid stresses on its surface and external forces (F) exerted on the bulk of the element. In contrast, the resulting velocity field (u) is ruled by the Navier–Stokes equation if the fluid is Newtonian, which means the viscosity of the fluid does not change with velocity. The small size of microchannels would keep the flow laminar in which the velocity of the droplet hydrogel in the fluid stream is not a random function of time. The impact of laminar flow is that two or more streams flowing in contact with each other will not mix except by diffusion, while under certain conditions, the height of the microchannel would cause nonuniformity in the streams, resulting in higher inertial forces at the interference point [51]. This might cause higher shear stress, which might affect both the volume of the droplets generated and the cells encapsulated within hydrogels [52].

The flow regime in microchannels is affected by several parameters such as capillary number, Weber number, and bond number. The capillary number helps determine the dominant flow regime in microchannels, which is defined as the viscous force (dynamic viscosity) to surface tension force in a fluid flow. A Weber number is a dimensionless number used in fluid dynamics to characterize the relative importance of inertial forces to surface tension forces in a fluid flow. When Ca << 1, viscous forces dominate, and the flow is typically characterized as a viscous-dominated or creeping flow. In this regime, flow is smooth and predominantly laminar. When Ca >> 1, surface tension forces dominate, leading to capillary-driven flow or inertial effects. The transition between these regimes can result in complex flow patterns. The capillary number affects the flow rate and velocity of fluids in microchannels. At low Ca, where viscous forces dominate, flow rates are relatively low, and velocities are slow. At high Ca, where surface tension forces dominate, flow rates can increase significantly, leading to faster velocities. The capillary number can also influence flow stability. In some cases, high Ca can lead to unstable flows, such as the formation of Taylor bubbles or droplets in microchannels. These instabilities can affect the mixing and mass transfer processes. However, when We << 1, surface tension forces dominate, and the flow is typically smooth, with laminar behavior. When We >> 1, inertial forces dominate, resulting in inertial or viscous inertial flow. The bond number is another dimensionless number used to characterize the relative importance of gravitational forces to surface tension forces in a fluid system. The bond number primarily governs the capillary rise in microchannels. When the bond number is much smaller than 1 (Bo << 1), gravitational forces are negligible compared to surface tension forces. In this regime, capillary action dominates, and fluids tend to rise or be drawn into narrow microchannels, creating capillary flow. The bond number also influences the wetting behavior of fluids in microchannels. When Bo << 1, fluids tend to wet the channel walls, resulting in good capillary flow and a continuous liquid front. Conversely, when Bo >> 1, gravitational forces are stronger, and the fluid may not wet the channel walls effectively, leading to partial or non-wetting behavior. The formulas needed to calculate these numbers are as follows:

Reynolds number: Re = (δ × ν × L)/η = Inertia force/viscous force,(1)

Capillary number: Ca = (η × ν)/γ = viscous force/capillary force,(2)

Bond number: Bo = (δ × g × L^2^)/ γ = Gravity force/capillary force, (3)

Weber number: We = (δ × ν^2^ × L)/γ = Inertia force/capillary force, (4)

where δ is the density of the flow (kg/m^3^), ν is the velocity of the fluid (m/s^2^), L is the characteristic length (m), η is the dynamic viscosity of the flow (Pa.s), γ is the interfacial tension (mN/m), and g is the gravity (m/s^2^).

The physics behind droplet generation in microfluidic devices is primarily governed by fluid dynamics, interfacial tension, and the geometry of the microfluidic channels. Droplet generation is commonly accomplished using a flow-focusing configuration, where a continuous phase, usually the oil phase, surrounds and constrains the dispersed phase, usually the aqueous phase, to form droplets. This configuration involves a main channel carrying the continuous phase and one or more side channels introducing the dispersed phase. The interfacial tension between the two immiscible fluids plays a crucial role in droplet formation. The interface between the two fluids tends to minimize its area because of capillary forces [53]. When the dispersed phase is introduced into the continuous phase through a side channel, the interfacial tension acts to deform the interface, leading to the formation of a droplet. Droplet size is influenced by the flow rates of both the continuous and dispersed phases. By controlling the flow rates, one can control the size and frequency of droplet formation. The ratio of the flow rates between the continuous and dispersed phases, known as the flow rate ratio, determines the size of the droplets produced [54]. The dimensions and geometry of the microfluidic channels also play a role in droplet generation. The width and height of the channels, as well as the shape of the junction where the dispersed phase is introduced, affect the droplet size and stability. Hydrodynamic forces, such as shear forces and pressure gradients, can help in focusing the dispersed phase into a narrow stream and promoting droplet formation [55]. These forces arise because of the different flow velocities and pressure profiles of the continuous and dispersed phases. In some cases, the fluids used in microfluidic devices may exhibit viscoelastic behavior, which can further influence droplet generation. Viscoelasticity refers to the combined characteristics of a fluid that exhibits both elastic and viscous properties [56]. The viscoelastic behavior can affect the droplet formation dynamics and stability. Therefore, optimizing the design and operation of microfluidic applications needs an understanding of the underlying physics of droplet generation in microfluidic devices.

## 3. Substrate Materials for Microfluidics

Lab-on-a-chip microfluidics is used in a wide range of laboratory applications such as separations [57], cell analysis [58,59], and microreactors [60]. The microfluidic fabrication process has employed a variety of materials that have been selected on the basis of their effects on the flow, absorptivity, biocompatibility, and function of microfluidic components [61]. The common materials used for microfluidics are inorganic (silicon, glass, and ceramics), rigid polymers (polyvinyl chloride and polymethyl methacrylate), and PDMS (polydimethylsiloxane) as organic material, among others.

### 3.1. Inorganic Materials

The development of a microelectromechanical system or MEMS is considered the pioneering work for subsequent fabrication techniques that include lithography, thin films, wet/dry etching, molding, and additive methods used in microdevices [48,62]. The materials selected for microfluidic device fabrication must adhere to photochemical properties, chemical resistance, and solvent compatibility [63]. For biological applications, these materials must also be biocompatible so that they can perform their function without eliciting toxic or injurious effects on cells and tissues. Silicon was the first material to be used in microfluidic fabrication since it is well characterized in semiconductor manufacturing [64]. However, glass was adopted later in the fabrication techniques because of its reliable electroosmotic flow, optical characteristics, resistance to organic solvents, and compatibility with biomolecules and cells [63].

#### 3.1.1. Silicon

Silicon is well known for its semi-conductivity properties where the surface modification characteristics, along with chemical properties and process compatibilities, make silicon an easy material for creating simple microfluidic devices. However, silicon has a high elastic modulus (~150 GPa), which makes the incorporation of active components such as valves and pumps in microfluidics much more complicated [65,66]. Furthermore, silicon is not optically transparent, which makes its devices unsuitable for mainstream fluorescence-based detection or direct fluid imaging [63]. The physical properties of silicon play a major role in hydrogel droplet generation in which its resistivity acts as a hidden key behind applying silicon in microfluidics. Silicon’s resistivity is higher than conductors and lower than insulators; therefore, it maintains an inversely proportional relation with temperature (Table 1) [67]. In addition, silicon accumulates less electrical charge on its surface and, therefore, generates weaker electrostatic forces, which make droplet generation more challenging compared to materials with lower resistivity. High thermoconductivity, stable electroosmotic mobility, and efficient handling of small fluid quantities are the key roles for using silicon in droplet-generating microfluidic devices [68]. However, one key application for hydrogel droplet generation is the encapsulation of cells, and because silicon is not optically transparent, imaging the cells requires the bonding of another transparent substrate and therefore diminishes its adoption. Nevertheless, silicon microfluidic devices have been extensively used in other biological applications. Pham et al. designed a silicon microfluidic device to accommodate solutions for immunogold silver staining by integrating self-assembled fluorescent microbeads and biological receptors into the designed device [69]. This approach enabled silver staining immunoassays with optical detection to measure the light absorption by the formed silver film on the microbead surface with captured antibodies.

#### 3.1.2. Glass

Chromatography and electrophoresis applications used glass fabrication for microfluidic devices with microchannels, flow reactors, and capillaries [70]. Glass is considered one of the most ideal device materials for bio-detection because of its optical transparency, low fluorescence background, surface stability, chemical resistance, and biological compatibility (Table 1) [48]. In contrast, glass fabrication techniques are not easy to implement as they require harsh neurotoxic etchants and require high temperatures to bond, complicating their ability to preload reagents before assembly [48]. To conduct experiments under high-pressure-driven flow, researchers used glass microfluidics as an approach to preventing peak dispersion. Gerhadt et al. fabricated a pressure-tolerant droplet microfluidic glass chip combined with gradient elusion reverse phase chromatography for that goal [71]. They found that the separation of analyte bands eluting from the high-pressure chip column is fractioned into numerous droplets in a continuous flowing oil phase. This approach prevented peak dispersion and facilitated post-column-processing of chromatographic fractions on a chip. For electrophoresis, glass chips have been used widely in microchip electrophoresis because of the electrical insulation and optical transparent properties of glass [72]. Mathies et al. developed integrated pneumatic valves and pumps that operate on the nanoliter scale, microfabricated resistive heaters and temperature sensors for integrated sample preparation, and integrated hydrogenated amorphous silicon photodiode detectors that enable point-of-analysis devices [73]. They mentioned that the development of fully integrated chemical and biochemical microprocessors will depend directly on the development of facile reliable integrated sample preparation technologies, as well as detection methods. The major outcome of their work is in providing evidence that a portable point-of-analysis lab-on-a-chip device is feasible [73]. Creighead et al. created nano-constrictions in fluidic channels that act as entropic barriers to DNA motion [74]. They used the size dependence of these entropic effects to separate DNA. They found that the length region of operation should be scalable with the choice of the constriction dimension to similarly separate larger or smaller DNA molecular sizes. They mentioned also that these fluid channels could be integrated with other functional devices such as in-plane optical waveguides for integrated fluorescent excitation and detection [74]. To develop an approach for the separation and detection of illicit drugs, Moreira et al. used a commercial glass microchip electrophoresis device that contained two pairs of integrated sensing electrodes for contactless conductivity detection [75]. It is shown that this process provided analyte concentrations of a limit of detection down to 40 µM. Other applications use glass as a material to generate and fabricate microfluidic devices such as femtosecond lasers [76,77,78], pumps, and valves [79] and for the additive manufacturing of microdevices [80,81] and cell analysis [82]. The advantages of using glass in microfluidics are its high transmittance, high processing accuracy, and processing methods that offer superior mass productivity, especially for hydrogel droplet generation. One of the superior characteristics of glass is that it generally provides a hydrophilic surface without pretreatment [83]. However, the major drawbacks of using glass in microfluidics are the low design freedom, difficulty in integrating dense and complex channel networks, and limited integration techniques for device coupling and interfaces [84]. The integration of liquid and dry reagents or hybrid components like filters is not feasible with glass microfluidics. Finally, the high material cost of glass makes it not preferable for hydrogel droplet generation [84].

### 3.2. Polymeric Materials

Thermoplastic or thermosoft plastic is a type of polymer that can be heated and softened using the heat-softened state such as thermoforming, or liquid state such as extrusion and injection molding [60]. Thermoplastics have been used widely in the mass production of high-quality devices since their introduction to commercial use in the 1930s [85]. The commercial applications of microfluidic devices use rigid polymers as bulk material for most of their productions.

The manipulation of polymethyl methacrylate (PMMA) in fabrication methods such as hot embossing, laser ablation, or precision milling has been extensively used in research studies because of its optical transparency [60]. This manipulation allowed for prototyping at small scales, which was found to be very useful in research studies [86]. However, for large-scale commercial production, the devices made by these fabrication methods are considered unsuitable because of the inherent variability [60]. Researchers found that the channel roughness can be high and deformation during the heated sealing process increases variability between devices. Photolithography is considered a good technique to avoid channel roughness, but it requires expensive cleanroom facilities and has a much slower prototyping cycle. Other microfluidic devices were fabricated using the most widely used materials such as polymethyl methacrylate (PMMA) [87,88], polycarbonate (PC) [87,89], and cyclic olefin copolymer (COC) [87,90] and moderately used materials such as polypropylene (PP) [91,92,93] and polyvinylchloride (PVC) [88]. Dolomite has introduced both glass and polymer droplet devices such as the two-reagent glass droplet chip, which can be used for generating droplets containing two reagents, and it is designed for a wide range of applications including high-throughput chemistry, biomedical analysis of cells, Janus particle formation, and polymerization research [94]. They introduced also a PDMS chip slide that is designed to be used with the PDMS chip interface to provide a flexible and fast solution for PDMS chip connections [95].

Polydimethylsiloxane or PDMS is a widely used polymer for the fabrication and prototyping of microfluidic devices. PDMS is a structure that contains carbon and silicon and is considered a mineral organic polymer [96]. As mentioned, PDMS is a silicone-based polymer that is generally opaque to ultraviolet (UV) light, which means it does not transmit or allow the passage of UV light, which has a wavelength range of 100 to 400 nm. PDMS is used as an insulating material in various applications such as fluidic systems; however, it is worth noting that the exact UV transparency of PDMS can depend on various factors such as the specific formulation, thickness, and processing conditions [97]. For the fabrication of microfluidic devices, PDMS is mixed with a crosslinking agent, poured into a microstructure mold, and heated to get an elastomeric replica of the mold. PDMS has the ability to perform with an appropriate host response in a specific situation and it is well known for its irreversible bonding to glass or another PDMS layer, which allows the production of multilayer PDMS devices via simple plasma surface treatment. Using spin coating, PDMS can be layered in a controllable thin thickness, which allows the fabrication of microchannels in multilayered devices [98]. It is a well-known gas-permeable material that enables cell culture by controlling the amount of gas through the material gas reservoirs. PDMS’s excellent oxygen permeability arises from its large number of repeating units of silicon, oxygen, and carbon, which form a flexible, cross-linked network that allows the transport of oxygen molecules through membranes, thus enabling on-chip cell respiration (Table 1) [99]. The large, flexible molecular structure of PDMS, combined with its low density and low surface energy, allows it to have a high permeability to gases such as oxygen. This property makes PDMS useful in various applications where gas permeation is desired, such as in biomedical devices/applications. Changing the composition or formulation of PDMS allows the easy modification of the oxygen permeability and makes the optimization for specific applications more flexible. The curing conditions, for example, can enhance the density, pore size, and surface energy of PDMS, which in turn affects its oxygen permeability. Lo et al. applied a multilayered microfluidic technique to integrate both aqueous- and gas-phase modulations via a diffuse membrane for temporal control of the oxygen microenvironment at the single islet level [100]. This technique creates a simulation sandwich around the microscaled islets within the transparent PDMS device that enables the monitoring of the glucose stimulus–secretion coupling factors via fluorescence microscopy. PDMS is widely used in bioengineering, tissue engineering, and microfluidic applications because of its biocompatibility, biostability, low toxicity, optical transparency, and versatile surface chemistry, as well as its mechanical flexibility and durability [101].

## 4. Fabrication Techniques for 3D-Printed Microfluidics

In contrast to traditional microfabrication that is based on photolithography, the new additive 3D fabricating of devices is a rapidly expanding technique for biomedical applications and microfluidic systems where complex three-dimensional structures are required with precise digital controls. The most relevant techniques used in microfluidics are stereolithography (SL), multi-jet modeling (MJM), and fused deposition modeling (FDM). Additionally, selective laser sintering (SLS) is at the top of these techniques, and it is widely used in manufacturing industries but cannot be used for microfluidics. SLS in microfluidics is not feasible since the powder precursor is very difficult to remove from small cavities [102]. The main goal of this section is to go through the fabrication techniques of 3D microfluidic devices used to create tissue on a chip to model diseases and to generate hydrogel droplets for tissue engineering and regenerative medicine.

### 4.1. Stereolithography

**Figure 2:**
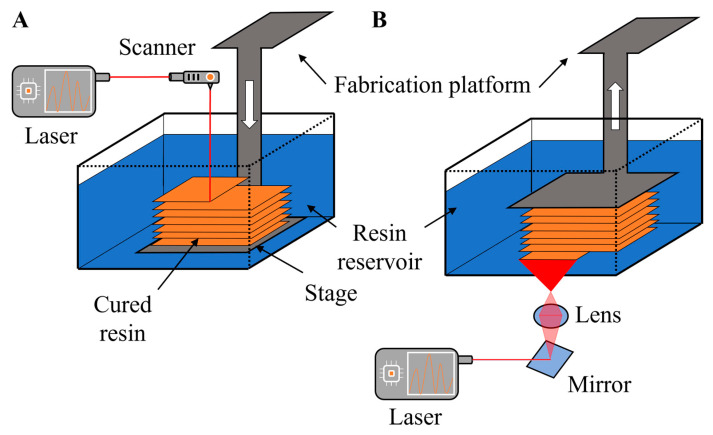
Stereolithography configurations. (**A**) The free-surface SL technique and (**B**) constrained-surface SL technique or the “Bat” configuration SL technique. Free-surface stereolithography (FSLA) and constrained-surface stereolithography (CSLA) are variations of stereolithography 3D printing. FSLA prints objects suspended within a liquid resin vat, making it suitable for complex, overhanging designs but requiring post-processing to remove supports. CSLA, on the other hand, constrains the object to a support structure within the resin vat, ensuring precise dimensional accuracy and minimal distortion, making it ideal for engineering and industrial applications.

Stereolithography (SL) fabrication creates a three-dimensional object layer by layer using selective light exposures to photopolymerize a precursor resin collected in a vat [103,104]. The digital sectioning of the 3D object into thin slices creates several layers, where each layer is considered as an image. The surface where resin treatment or photopolymerization of resin happens divides SL into two different approaches (Figure 2) [105], the free-surface and constrained-surface approaches. In the free-surface approach, the top surface exposed to air is subjected to a laser to photopolymerize the resin. Then, the metal build stage is always submerged in a resin vat where it is translated downward into the vat after every layer is printed (Figure 2A) [102]. However, the most advanced SL approach is the constrained-surface approach. The resin in this approach is photopolymerized against the bottom surface of the vat and not the top one like in the free-surface approach. The building of layer by layer by photopolymerization happens by suspending the metal build plate upside down above the vat before being separated from the bottom surface of the vat [102]. The bottom surface is usually coated with PDMS and later replaced by flexible PTFE films for easy separation, and the metal build plate is then returned to its original suspended position. This process is called the “Bat” configuration, where an upside-down orientation is followed to build the final object (Figure 2B) [106].

The major drawback of bat configuration is in the mechanical separation setup where it can induce stress fractures or bending of delicate features and increase roughness between layers [106]. The vat depth in the free-surface approach limits the object depth, while the height in the bat configuration is limitless. The oxygen in these experiments works as an inhibitor for the process of photo-polymerization, and thus, the time of curing in the bat configuration is faster than that of the free surface because of the reaction that happens away from the air–resin interface [102]. Some modifications to the constrained surface technique subjected the bottom plate to oxygen and utilized controlled oxygen inhibition to prevent the last cured resin from adhering to the bottom plate. Because of this modification and because of the suspension of the last photo-polymerization in resin, separation of the bottom part from the built part is no longer needed, and thus, the printing speeds can be increased almost 100 times in the continuous printing approach [107].

**Figure 3:**
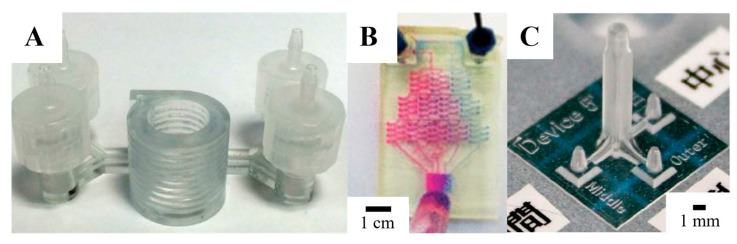
Microfluidic devices printed with SL. (**A**) Spiral microchannel with trapezoid cross-section used for size-selective separation of bacterial cells. This fabricated device incorporates 10 loops of helical microchannels to ensure the required length for particle migration. The trapezoidal channel cross-section has a width of 1000 µm, with inner and outer heights measuring 250 µm and 500 µm, respectively. These dimensions were determined by the minimum resolution capabilities of the 3D printer used for device fabrication, and the experimental results demonstrate its satisfactory performance. Both the inlet and outlet are situated on the same plane. However, the inlet connects to the top of the helix via a vertical channel. This arrangement was chosen to avoid overhanging structures, which are not conducive to 3D printing. To facilitate a secure seal and prevent leakage, the inlets and outlets were connected to tubing using barbed luer-lock connectors [110]. (**B**) A complex microfluidic mixer and gradient generator printed with a commercial desktop SL system. The device consisted of three distinct fluidic layers, each designated for sample handling, reagent delivery, and the addition of nitrite standards. It also featured micromixers and a series of detection cells with varying depths ranging from 0.5 to 15 mm. This depth variation was implemented to extend the linear range of the colorimetric assay. Remarkably, this intricate microdevice was 3D printed in less than 5 h and was successfully employed to analyze nitrate levels in tap water, producing results consistent with those obtained through an off-chip assay. The cost-effectiveness of the 3D printer used, combined with the ease of creating intricate 3D microfluidic devices, holds significant promise for advancing the development of microfluidic applications [111]. (**C**) Three-dimensional flow channels for preparing double emulsions. The device features three cylindrical channels that are coaxially aligned and oriented perpendicular to the substrate. The dimensions of the device fall within a compact 1 × 1 cm^2^ footprint, with the internal diameters of the inner, middle, and outer channels configured at 50, 500, and 1000 μm, respectively [112]. Panels (**A**–**C**) are reproduced from [110,111,112] with permission of the Springer Nature, American Chemical Society, and Elsevier, respectively.

Microchannels in stereolithography are built by photo-polymerizing the channel walls and removing the uncured resin from the channel cavity once the printing is finished [108]. Several studies used SL in microfluidics such as immunomagnetic separation of bacteria [109], separation of cells by using helical channels with trapezoid cross-sections (Figure 3A) [110], gradient generation (Figure 3B) [111], emulsion droplet generators (Figure 3C) [111,112], and DNA assembly [113]. The type and viscosity of resin play a major role in the minimum desired cross-sectional area of a microchannel, in addition to laser spot size or pixel resolution [114]. Wang et al. fabricated a multilayer microfluidic droplet generator device using the liquid-crystal-display-based stereolithography 3D printing system. They found that the 3D-printed flow-focusing droplet generator worked properly and could generate droplets with sizes between 50 and 185 mm^2^ [115]. Kamperman et al. engineered a 3D parallelized microfluidic droplet generator with equal flow profiles by computational fluid dynamics for designing and stereolithography for printing [116]. They reported that droplets and micromaterials of different compositions and complexities were produced, including solid microgels and hydrogel microcapsules. The production of monodispersed microdroplets with diameters between 150 and 1000 µm by tuning the water/oil flow ratio was achieved [116].

### 4.2. Multi-Jet Modeling

Multi-jet modeling (MJM) is a 3D printing technique where layers are built on a tray on top of each other via an inkjet head, which delivers a curable liquid photopolymer that can be rapidly polymerized by UV. For some applications when the printed shapes need support, the inkjets can deliver supportive material that can be dissolved after finishing the print [102]. MJM is known for its ability to build an object with multiple materials such as hard and soft plastics and elastomers [117]. However, these materials are expensive, and studies are still investigating their biocompatibility and biofunctionality [102]. MJM is widely used in the medical field where anatomically accurate models were printed for orthopedic [118], cardiac [119], and intracranial surgeries [120].

**Figure 4:**
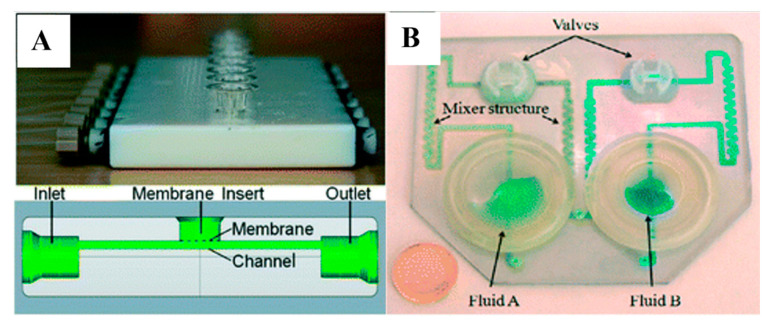
Microfluidic devices printed with MJM. (**A**) A microfluidic device printed with an Objet Connex 350 using Vero white plus as the resin, incorporating adapters for syringes, 8 channels, inlets, outlets, and a port for inserting a polycarbonate membrane for cell culture [102,121,122]. (**B**) A fluid mixer and homogenizer printed with an Objet Eden 250 using the Full Cure 720 resin with a 375 µm square channel. An electrode was seamlessly integrated into the wall jet configuration of the device for the purpose of electrochemical detection of catechol [102,123,124]. Panels (**A**,**B**) are reproduced from [121,123] with permission from the American Chemical Society and Elsevier, respectively.

MJM printing has a high resolution and multi-material printing capability, which makes it an attractive approach for microfluidic applications [102]. However, to create arbitrary microfluidic channel networks, a sacrificial material must be used that can reliably be removed and cleared from an enclosed channel. To study the transport and pharmacokinetic profiling of drugs, MJM-printed flow channels are integrated with semi-permeable membranes to support cell cultures (Figure 4A) [121,122]. Stratasys released a soluble support material that is dissolvable with NaOH, which is considered an option for creating smaller microchannels; however, the removal setup is limited only to diffusion [102]. An Objet Eden printer was used to print a microfluidic mixer and homogenizer [123] with a microfluidic channel of 375 µm square using full cure 720 resin and integrated with an electrode in the wall jet configuration for electrochemical detection of catechol (Figure 4B) [124]. However, MJM printings/models are well known for their poor mechanical properties and lower temperature resistance, which makes them unsuitable for applications in microfluidics such as droplet generation, especially when dealing with encapsulated cells. In addition, MJM is still considered an expensive technique for 3D fabrication, which explains its limited use in microfluidics. Despite that, Chen et al. generated sodium alginate microspheres using a 3D-printed reconfigurable high-fluid-pressure modular microfluidic system [125]. They fabricated all the modules using a 3D printer based on multi-jet modeling (MJM) technology. A single droplet and dual droplets were generated using the assembled microfluidic system, in which they reported that the flow rate of calcium chloride was inversely related to the length of the sodium alginate microspheres [125].

### 4.3. Fused Deposition Modeling

Fused deposition modeling (FDM) or thermoplastic extrusion is a 3D printing technique where the thermoplastic material is heated and extruded from a motor-driven nozzle. After extrusion, the material hardens immediately via spontaneous cooling; however, the alternating layers need to be fused to provide structural strength in the vertical orientation [102]. To overcome this challenge, a heated enclosure is incorporated into the printing machine to increase the layer fusion and structural integrity, but this cannot completely eliminate the material anisotropy [126]. The gamma irradiation technique was employed in the FDM process to promote crosslinking between layers, which resulted in an increase in the intra-layer strength of FDM-printed objects [127]. These strategies have been employed in several applications where hydrogels, cell-laden solutions, metallic solutions, etc. were extruded through a nozzle head to create batteries [128], interconnects [129], and electrodes within biological tissue [130].

**Figure 5:**
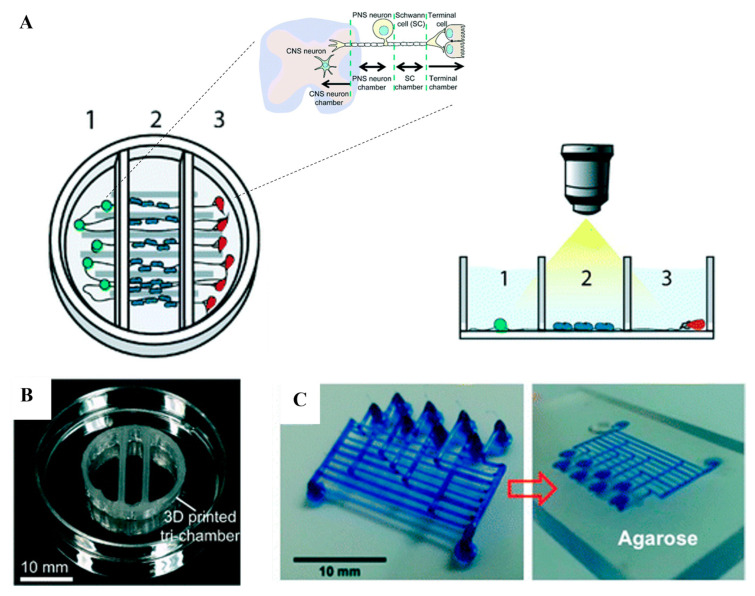
Microfluidic devices printed with FDM. (**A**) Schematic of customized 3D-printed nervous system with 350 µm wide printed microchannels on a chip (3DNSC) for the study of viral infection in the nervous system showing (1) PNS neurons in chamber 1, (2) Schwann cells in chamber 2, and (3) terminal cell junctions in chamber 3. The Schwann cells and the terminal cells interact with the neurons and each other solely via the axonal network [132]. (**B**) Image of the 3D-printed customized nervous system on a chip device. A 3D nervous system on a chip showing a perpendicular assembly of the microchannel and tri-chamber components [132]. (**C**) FDM carbohydrate scaffold surrounded with the live-cell-laden extracellular matrix. The carbohydrate dissolves in agarose hydrogel and microchannels appear [133]. Panels (**A**,**B**) are reproduced from [132] and panel (**C**) is reproduced from [133] with permission from the Royal Society of Chemistry.

Microfluidic fabrication with FDM is challenging because of the lack of structural integrity between the printed layers, which causes weak seals. The size of filaments and nozzles that are larger than the size of the microchannel used in microfluidics is another challenge [102]. Quero et al. used FDM printers to print microchannels with sizes of less than 100 µm [131]. They found that the vibrational effects, materials of the printing bed, and nozzle diameter are the most important parameters to consider for good transparency and layer uniformity of the prints. Johnson et al. used an FDM printer to customize a 3D-printed nervous system with 350 µm wide microchannels on a chip (3DNSC) for the study of viral infection in the nervous system (Figure 5A) [132]. Micro-extrusion 3D printing strategies enabled the assembly of biomimetic scaffold components such as microchannels and compartmented chambers for the alignment of axonal networks and spatial organization of cellular components (Figure 5B). FDM is also used for generating 3D microfluidic molds in a sacrificial material that can be dissolved after the bulk material is infiltered, and thus, the 3D replica molding is assembled [102]. Miller et al. created an endothelial-cell-lined vascular network embedded in engineered tissue. Using FDM printing, a sacrificial carbohydrate scaffold was printed and surrounded with a live-cell-laden extracellular matrix. The sugar was then dissolved with cell culture media and the voids were seeded with endothelial cells (Figure 5C) [133]. Another group employed the same concept by printing a microfluidic channel network in agarose by using isomalt, which is a sugar alcohol, as a sacrificial material that can be dissolved by agarose hydrogel [134].

FDM is used widely in several applications in microfluidics for its high reliability, low cost, and simple operation [135]. The major drawbacks of using the FDM technique are the relatively poor resolution and rough surface finishing, which limit its applications in droplet microfluidics [136]. Several applications have used FDM for droplet microfluidics such as the printing of T-junction and flow-focusing structures for droplet creation [137,138]. The large size of the nozzle and filament make the printed channel large, despite the high-resolution capabilities of the motion system, which make the printable minimum channel dimensions larger than the designed ones [135]. To overcome this challenge and make this technique applicable in droplet microfluidics, Gale’s research group [139] reduced the channel width down to 40 µm by lowering the nozzle to the print surface, which flattened the extrusion lines, and thus, the line width increased. They reported that the channel width was smaller than the designed width by 100 µm [139].

Using stereolithography, multi-jet modeling, and fused deposition modeling for 3D-printed microfluidics introduces several limitations concerning the channel sizes of the printed devices. This section of the paper aims to compare the resolution of the channel dimensions between lab-scale and commercial-scale implementations for the three reviewed techniques. Table 2 represents the results, revealing that the lowest resolution was obtained using stereolithography (18 µm × 20 µm) with a custom resin on a lab scale. It is worth noting that there have been relatively few studies employing multi-jet modeling in microfluidic fabrication, primarily because of its high cost in academic labs. Both stereolithography and fused deposition modeling achieved lower resolutions on a lab scale when compared to commercial scaling (Table 2 and Table 3).

## 5. Biomaterials for Hydrogel Droplet Generation

Various biomaterials have been developed in cell culture and tissue engineering based on microfluidic devices. Alginate [142], chitosan [143], GelMA [144,145], hyaluronic acid [146], and poly(ethylene glycol) diacrylate (PEGDA) [146] hydrogels can provide a tunable environment for cells and support their adhesion, growth, and proliferation. PEGDA is a type of hydrogel precursor that can be used in the generation of hydrogel droplets. Despite the potential advantages of using PEGDA in hydrogel droplet generation that is not limited to biocompatibility, high water content, control over droplet size, UV-crosslinking, and versatility, its major drawbacks limit its usage in so many biomedical applications [147]. The disadvantages of using PEGDA in hydrogel droplet generation include limited mechanical strength, limited control over network structure, susceptibility to water loss, UV exposure requirements, and potential toxicity [148]. However, these disadvantages can be addressed through careful selection of parameters and other droplet precursors. Other drawbacks are the cell adhesion and protein deposition that are not supported by PEGDA. For this reason, this section will discuss only the applications of alginate, chitosan, GelMA, and hyaluronic acid in 3D cell cultures based on microfluidics.

### 5.1. Alginate

**Figure 6:**
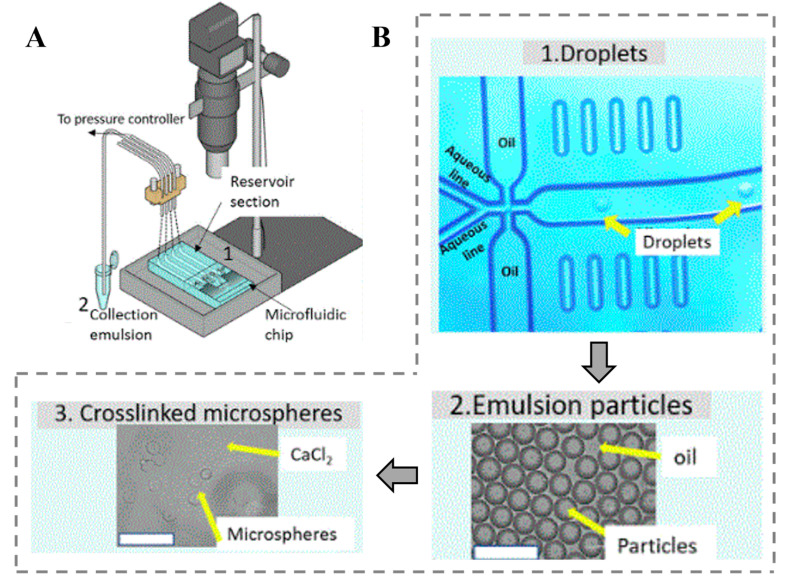
Schematic representation of the air-pressure-driven microfluidic system [154]. (**A**) The air-pressurized system aids the injection of the phases located within the reservoir toward the two-reagent flow-focusing microfluidic chip. (**B**) The process of formation by 1: droplets formed inside the microfluidic chip, 2: emulsion particles collected at the microtube, and 3: crosslinking emulsion particles ex situ. Panels (**A**,**B**) are reproduced from [154] with permission from Elsevier.

Alginate is a naturally anionic polysaccharide mainly found in brown algae and has been extensively investigated and used in different biomedical applications. Alginate is biocompatible and biodegradable with relatively low cost and mild gelation abilities by the addition of divalent cations such as calcium ions [149]. Various crosslinking methods can be applied to prepare alginate hydrogels where they have wide similarity in structure to the extracellular matrices of living tissues. The applications of alginate hydrogels are not limited to wound healing [150], delivery of bioactive agents such as small chemical drugs and proteins [151], and cell transplantation [143]. The physical properties play a significant role in controlling the stability of alginate gels, the rate of drug release from gels, and the phenotype and function of cells encapsulated in alginate gels. These properties can be enhanced by increasing the molecular weight of alginate, but the high molecular weight makes the solution more viscous, which is often undesirable in processing (Table 4) [152]. Mixing high-viscosity alginate solution with proteins or cells could cause damage to the crosslinking matrices and the cells because of the high shear forces generated during mixing and injection into the body [153]. Several applications used alginate microspheres for the encapsulation of bioactive molecules. Aguilar et al. [154] formed alginate microspheres by optimizing microfluidics parameters for high encapsulation of bioactive molecules where one of their main goals is to test if the microspheres produced were detrimental to biological environments in vitro. To investigate more, an air-pressure pump-controlled system was used to prepare the alginate microspheres. Figure 6 shows the developed and characterized alternative encapsulation and crosslinking approach for allowing the integration of bioactive molecules. They found that the flow rate and polymer concentration parameters can be optimized to increase the encapsulation efficiency of bioactive molecules into alginate particles without varying the pH. Singh et al. [155] introduced a method for developing magnetic alginate microparticles (MAMs) using a microfluidic platform for magnetically templating hydrogels. They found that this approach allows control over the magnetic iron oxide loading of the MAMs through iron quantification [155]. However, to improve the shape fidelity of alginate, researchers are mixing alginate with GelMA to achieve high 3D printability. Chen et al. [156] investigated the synthesis and mechanical characterization of a gelatin methacrylate–alginate (Gel-Alg) composite hydrogel. They reported that Gel-Alg is capable of tuning its viscoelastic strain and elastic recovery properties and can be potentially used to design ECM-mimicking hydrogels [156].

### 5.2. Chitosan

Chitosan is a linear polysaccharide composed of randomly distributed β-linked D-glucosamine and N-acetyl-D-glucosamine [157,158]. It is known as a sugar that comes from the chitin shells of shellfish like crab, lobster, and shrimp. To describe the characteristics of chitosan, the degree of deacetylation and molecular weight are the elements to consider, varying depending on the source and process of production [159]. Chitosan has been used widely in biomedical applications for the last two decades for its suitability in cell culture, biodegradability, osteoconduction, porous structure, ease of modification and processing, and biocompatibility (Table 4) [160]. Some of the biomedical applications of chitosan are not limited to cartilage tissue engineering [161], wound healing [162], drug delivery systems [163], and orthopedic applications [160]. Chitosan is used widely in microfluidics, which is considered the most promising platform to fabricate high-performance chitosan-based multifunctional materials with monodispersed size distribution and accurately controlled morphology and microstructures. Microfluidic technology has precise fluid control, various shapes of the microchannel, and a multichannel programmed mixing process that aids the synthesis process of various types of chitosan with particle morphology and uniform particle-size distribution [164].

To generate chitosan microspheres, the Y-type microfluidic device has been used by Wang et al. [165]. However, to get strong electrostatic interaction, chitosan and poly acrylic acid (PAA) were mixed to form CS-PAA, and then, CS-PAA droplets were prepared using a Y-type device with the dispersed phase of 1.5 wt% chitosan, 0.1 wt% poly acrylic acid, and 40 wt% acetic acid [166]. To effectively reduce the diffusion, evaporation, and reactions of inside actives with the external environment, microencapsulation was implemented. The fluid interfacial tensions could be used, too, in microfluidic technology to form highly controllable emulsions for preparing controllable microcapsules [167]. Chitosan has been used widely in building blocks for constructing nanoparticles because of its superior biological properties [168]. However, the hydrophilic nature of chitosan makes it appropriate for holding hydrophilic drugs while causing inadaptability for loading hydrophobic drugs [169]. To facilitate the fabrication of hybrid chitosan nanoparticles, cellulose laurate (CL) was added to the chitosan solution to improve the encapsulation capacity of hydrophobic drugs [164]. In particular, achieving an adjustable micro-sized compartmental internal chamber in microfibers is essential for improving the chitosan functions in the biomedical engineering field. Therefore, combining microfluidic technology with excellent manipulation of microflows with chitosan would enhance the biofunctional manufacturing of microfibers with controllable structures [170,171]. Chitosan-based membranes have been used widely in different applications such as extraction [172], separation [173], dialysis [174], and tissue engineering [175] because of their excellent performance. Chitosan-based membranes can be developed following both laminar-flow-based interfacial reaction technology and hydromechanical focusing and laminar flow interface reaction.

### 5.3. GelMA

Gelatine methacryloyl (GelMA) has been used widely in biomedical applications and tissue engineering for its biocompatibility and tuning ability of mechanical properties [176]. The suitability of GelMA in these applications was tested extensively by characterizing the physical properties such as porosity, elastic modulus, degradation, and water swelling and by investigating the cell response parameters like cell viability, proliferation, differentiation, and spreading (Table 4) [177]. Manipulating the synthesis and processing of GelMA, such as the conditions of crosslinking, offers versatility in tuning its characteristics. Subjecting the GelMA hydrogels to cryogenic treatments such as freeze drying would generate porous scaffolds with controlled pore sizes and porosity [178]. Van Vlierberghe et al. identified the preparation of porous GelMA hydrogels following the cryogenic treatments of the chemically crosslinked hydrogels [179]. They reported the successful preparation of GelMA hydrogels with gradient pore sizes using a gradient cooling rate strategy. They also showed that the average pore sizes of prepared hydrogels were inversely related to the concentration of GelMA solution and cooling rate [179,180]. The relationships between chemical, physical, and bio-responsive characteristics of GelMA hydrogels were reported. Chen et al. reported that the compressive modulus of GelMA hydrogel was directly proportional to the degree of methacryloyl substitution [181]. Nichol et al. [182] showed the directly proportional relationship between the compressive modulus of GelMA and the mass/volume fraction. They also reported that the swelling ratio decreased with increased methacryloyl substitution and the GelMA mass fraction [182]. The impact of the mass fraction of hydrogels on cell proliferation was also investigated, and they found that the proliferation was inversely proportional to GelMA mass fraction [182].

**Figure 7:**
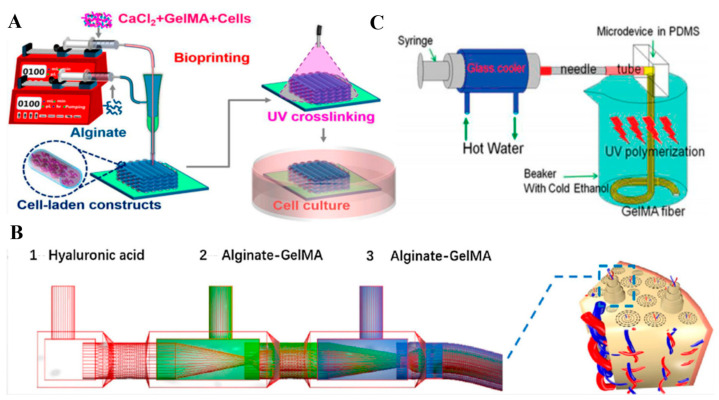
A schematic diagram showing the (**A**) extrusion-, (**B**) laminar-flow-, and (**C**) electrospinning-based methods in fabricating GelMA hydrogel microfibers. (**A**) Extrusion method: GelMA served as the core, while alginate served as the sheath to support and confine the GelMA hydrogel in the core to allow the subsequent UV crosslinking [186]. (**B**) Laminar-flow-based method: a double coaxial laminar-flow-based strategy to construct double-layer hollow microfibers that could mimic the native osteon [187]. (**C**) Electrospinning method: micro-structured patterns to generate GelMA hydrogel fibers [188]. Panels (**A**–**C**) are reproduced from [186,187,188] with permission from the Institute of Physics Publishing, Elsevier, and Wiley, respectively.

GelMA hydrogels were used in several applications as raw materials for tissue engineering building blocks, such as microfibers and bioink for complex structures in microfluidic bioprinting platforms [183]. Microfibers have been extensively used in biomedical applications in the field of tissue engineering, 3D culture, and cell encapsulation [184,185]. Extrusion, laminar flow, and electrospinning-based methods are the three methods followed in microfluidics for fabricating GelMA hydrogel microfibers [183]. The extrusion method involves extruding pre-gel solution into a gelator solution using a syringe needle. Liu et al. used this method to fabricate GelMA/alginate microfibers [186]. GelMA served as the core to provide a favorable 3D microenvironment for cells, while alginate served as the sheath to support and confine the GelMA hydrogel in the core to allow the subsequent UV crosslinking (see Figure 7A) [186]. In the laminar flow, the pre-gel solution and gelator solution would form a coaxial or parallel flow within a microfluidic channel [183]. Liu et al. [187] modeled a double coaxial laminar-flow-based strategy to construct double-layer hollow microfibers that could mimic the native osteon (see Figure 7B). The mixed bioink (alginate and GelMA) was used in this work to obtain an enhanced mechanical strength and prominent bioactivity [187]. Electrospinning is the most-used method in generating GelMA hydrogel microfibers [183]. Using microfluidic spinning, the GelMA hydrogel fibers were generated with micro-structured patterns (see Figure 7C) [188]. They reported a successful printing of uniform and well-arranged grooves on the fabricated microfibers. These grooves could efficiently facilitate cell encapsulation and adhesion, in addition to being used as templates for the creation of fiber-shaped tissues or tissue microstructures [188]. GelMA was used as bioink in several applications in tissue engineering based on microfluidic technology [189]. A 3D-bioprinted multiscale scaffold integrating the 3D micro- and macroenvironment of native nerve tissue based on GelMA/chitosan microsphere (GC-MSs) modular bioink was presented by Chen et al. [190]. It was reported that the nerve cells within a 3D environment were protected by the GC-MSs-based 3D bioprinted composite scaffold [191]. GelMA hydrogels were used as simulation units in tissue engineering for several applications not limited to scaffolds for 3D cell culture and components for organ-on-a-chip [182]. A flow-focusing microfluidic device was utilized for generating core-shell GelMA microgels with highly controllable sizes [191]. These microgels were found to provide a suitable cellular microenvironment that could be used as an in vitro platform to culture cardiac side population (CSP) cells on the surface, where they migrated and spread well onto their cell-conductive surroundings [191]. The other application of GelMA hydrogels in tissue engineering is organ-on-a-chip, which refers to a bionic system that simulates the in vivo microenvironment of living organs and provides more in vitro models related to the physiology of human organs [192,193]. A vessel-on-a-chip system based on GelMA hydrogels was presented by Nie et al. [192]. This study reported that viable attachment and uniform spreading of human umbilical vein endothelial cells (HUVECs) on the surface of the inner wall of the channels achieved a good survival rate [192]. GelMA droplet generation poses a great challenge, especially with microfluidics, while becoming more attractive owing to its remarkable biocompatibility for cellular functionalization and rapid crosslinking ability. GelMA microdroplets with low concentration have been reported to be more appropriate for cell studies because of their larger pores while showing superior elasticity and an enhanced degradation profile. Xie et al. showed that the low concentration GelMA microdroplets (5% *w*/*v* GelMA) printed with electro-assisted bioprinting provided a suitable microenvironment for laden bone marrow stem cells, which showed great potential use in cell therapy [193]. Mohamed et al. developed a microfluidic flow-focusing platform for on-chip fabrication and filtration of micro-GelMA droplets [194]. Cell viability of the encapsulated cells was found to be around 85% on day 1 and was maintained throughout 5 days.

### 5.4. Hyaluronic Acid

Hyaluronic acid (HA) is a nonsulfated glycosaminoglycan composed mainly of repeating polymeric disaccharides of D-glucuronic acid and N-acetyl-D-glucosamine linked by a glucuronic β bond [195,196]. HA polymers are well known for their variety of physiochemical properties in which they occur in different configurations and shapes that are directly related to their size, salt concentration, and pH (Table 4) [197]. Hyaluronic acid, unlike other GAGs, may form aggregates with proteoglycans while not covalently attached to a protein core [198]. One of the advantages of using HA in biomedical applications is the enclosure of a large amount of water, making the solution highly viscous even at low concentrations [199]. Hyaluronic acid is found in all tissues of the body such as skeletal tissues [200], heart valves [201], and the lungs [202], where it is produced primarily from mesenchymal cells [201]. Several studies have shown the functional role of HA in molecular mechanisms and indicated the potential role of HA in the development of novel therapeutic strategies for many diseases [203]. It is reported that during tissue injury and wound healing, the HA starts to synthesize before regulating several aspects of tissue repair such as the activation of inflammatory cells to enhance the immune response [204,205]. HA is also known to be involved in tumor progression by providing the framework for blood vessel formation and fibroblast migration [206,207]. Chiesa et al. presented a microfluidic technique to obtain lipid- and hyaluronic-acid-based nanoparticles for protein delivery with high batch-to-batch reproducibility, precise modulation of the size of the nanoparticles, improved drug encapsulation efficiency (EE), and easy scale-up [208,209]. This method has been reported to provide a direct and rapid production pathway for nanomedicine from a bench to a prototype product. In addition, it has been shown to effectively load proteins, which develops a lot of formulations for protein-based therapies [210]. Russo et al. [211] proposed a microfluidic platform that takes advantage of interferences caused by the presence of gadolinium diethylenetriamine penta-acetic acid (Gd-DTPA) in flow-focused nanoprecipitation, which enables the formation of monodispersed crosslinked hyaluronic acid nanoparticles (cHANPs) under 100 nm to impact the relaxation rates of Gd-DTPA. It was mentioned that the designed strategies have allowed the fine control of the monodisperse average size of 40 nm, surface charge, and loading capability of 59% [211].

The implementations of alginate, chitosan, GelMA, and hyaluronic acid in droplet microfluidics are discussed in the following. The major advantages of alginate are its biocompatibility, mechanical properties, and fast gelation kinetics, in addition to the low cost. However, the major drawback is the poor dimensional stability, which can be resolved by mixing with other biomaterials such as gelatin or GelMA. Chitosan shares the same advantages as alginate such as nontoxicity, biocompatibility, biodegradability, and ease of preparation. However, it has wider applications, especially in medicine, with a major drawback, which is the low solubility in aqueous preparations. GelMA has been used widely in biomedical applications, especially cartilage tissue regeneration, based on its mechanical properties, which could simulate the natural cartilage matrix. GelMA’s biocompatibility made it an extraordinary candidate for stem cell adhesion, proliferation, and chondrogenic differentiation. Hyaluronic acid is the main component in extracellular matrices of articular cartilage tissues and other tissues in the human body. It is well known for its physiochemical properties, which play a key role in droplet microfluidic generation.

Utilizing biomaterials for hydrogel droplet generation is widely recognized as a challenging process that demands meticulous handling and precise control. In this study, we assess the droplet sizes produced from alginate, chitosan, GelMA, and hyaluronic acid, comparing them to the techniques mentioned earlier (refer to Table 5). It is important to highlight that there is a scarcity of research on multi-jet modeling and fused deposition modeling of GelMA and hyaluronic acid microdroplets, primarily because of the difficulties involved in handling these materials and the high cost associated with these techniques.

## 6. Generating Hydrogel Droplets in Microfluidics

Several methods have been used to generate hydrogel microparticles where emulsification [216,217], direct agitation and grinding of the polymer [218], and microfluidics [219] are the most widespread methods applied. Microfluidics has shown several unique advantages compared to other alternative methods in which the size and shape of the microparticles can be precisely controlled, allowing the generation of a large variety of shapes and compositions of the monodispersed microparticles [220]. Microfluidic devices have also shown their suitability in controlling reaction, heat, and mass transport rates, reducing waste time, and enabling the control of the characteristics of the microparticles [221]. For the production of microparticles, multiphase systems are applied that comprise a continuous phase that is usually an oil and a dispersed phase, which is a hydrogel precursor in our review and a surfactant [47]. The driving forces for the generation of droplets are shear stress and the interfacial tension between phases. Surfactants prevent the coalescence of the droplets and lower the interfacial tension between the phases [221,222]. However, the final size and shape of the microparticles depend on multiple parameters, including flow rates of fluids, geometry, material composition, size of the channels, and fluid properties [220].

### 6.1. Mechanics of Droplet Generation

Several techniques have been introduced to produce hydrogel droplets such as dielectrophoresis-driven droplet generation [223], electrowetting on dielectric-driven droplet generation [223], electrohydrodynamic manipulation [223], thermocapillary manipulation [223], magnetic actuation [224], acoustic actuation [225], and hydrodynamic manipulation at the microscale level [226]. However, hydrodynamic manipulation is the most used method for microfluidic droplet production. Hydrodynamic droplet generation techniques use the shear and interfacial forces to generate droplets, where as mentioned before, the production size, rate, and shape of the droplets are dependent on the flow rates, geometrical modeling of microchannels, and physical properties of the phases [220]. PDMS is the most widely used material in the fabrication of microchannels using hydrodynamic manipulation because of the low-cost fabrication process and easy control of surface chemistry [220]; however, PDMS swells or degrades when exposed to some solvents [227]. In contrast, other materials have been introduced to replace PDMS, such as glass and silicon, because of the reasons mentioned in the previous sections [228].

**Figure 8:**
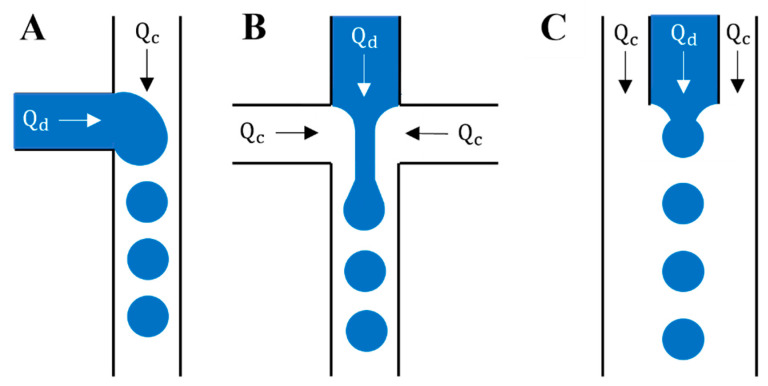
Schematic diagram of the flow patterns in droplet microfluidics. (**A**) T-junction scheme, (**B**) flow-focusing scheme, and (**C**) co-flow scheme. Q_c_ is the continuous phase, and Q_d_ is the dispersed phase. T-junction, flow focusing, and co-flow are distinct microfluidic configurations used for different fluid manipulation purposes. T-junction involves the perpendicular intersection of two fluid streams to achieve rapid mixing at a single point and is commonly used for applications requiring immediate reaction or reagent mixing. Flow focusing, on the other hand, entails the co-flow of immiscible fluids, where one fluid surrounds and controls the flow of another, often leading to droplet or particle generation. It provides precise control over droplet size and encapsulation, making it suitable for applications like microencapsulation and single-cell analysis. Co-flow involves the simultaneous flow of multiple fluids in the same direction within a channel, allowing for controlled mixing or diffusion over an extended length, which is advantageous in applications such as chemical reactions and drug delivery. Each scheme is chosen based on specific requirements for fluid manipulation and mixing in microfluidics.

The most common designs for the production of microdroplets are T-junction, co-flow, and flow-focusing geometries [229]. For the T-junction configuration (Figure 8A), the main channel has the continuous phase, while the secondary channel has the dispersed phase [47]. The flow of the dispersed phase in the main channel in the flow-focusing configuration is squeezed by the continuous phase entering from the two lateral channels (Figure 8B) [230]. The co-flow configuration has a similar configuration to the flow-focusing configuration, where the dispersed phase flows in an inner flow parallel to the outer continuous phase (Figure 8C) [229]. Different parameters directly affect the size of droplets such as fluid density and viscosity, surface tension, flow rates, surface properties, and the geometry of the device [229]. The dimensional analysis of the nondimensional size of the droplets, capillary (Ca), and Reynolds (Re) numbers of the dispersed and continuous phases, viscosity ratio, and contact angle led to a correlation [47]. This correlation showed that viscous forces are dominant, and no significant flow variation has been shown when the Reynolds number changes [223], while with the high-speed flow in microchannels, the inertial forces become significant, and the Reynolds number effect should be taken into consideration [231]. Zhao et al. [232] reported a novel method using capture antibodies immobilized on porous poly(ethylene glycol) diacrylate (PEGDA) hydrogel microspheres to enable high-sensitivity VEGF detection in arrayed microfluidics. Their method used the flow-focusing scheme for antibody encapsulation, trapping, and flow perfusion on a single device [232]. Duan et al. [233] achieved a multiplexed detection panel for diabetes antibodies targeting insulin, GAD, and IA-2 using flow-focusing scheme droplet microfluidics. They reported that the serpentine microfluidics achieved spatial multiplexing of the microgels that avoided issues associated with spectral- or imaging-based techniques. In addition, they found that their microfluidic detection panel has the potential to improve diabetes, as well as to investigate immunogenic mechanisms in diabetes [233]. Chen et al. [179] presented a 3D bioprinted multiscale scaffold integrating the 3D micro- and macroenvironment of native nerve tissue based on GelMA/chitosan microsphere modular bioink (GC-MSs). The GC-MSs provided a similar mechanical property to nerve tissue for nerve cell proliferation and differentiation. The round shape of the GC-MSs was beneficial for cellular adhesion and proliferation of cells.

**Figure 9:**
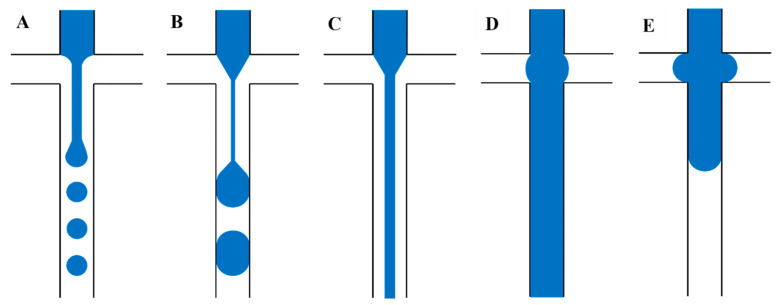
Schematic diagram of the different flow regimes in a flow-focusing device: (**A**) jetting, (**B**) dripping, (**C**) threading, (**D**) tubing, and (**E**) viscous displacement. Jetting, dripping, threading, tubing, and viscous displacement are various techniques used in microfluidics to manipulate fluid flow and droplet formation. Jetting is characterized by forceful ejection, resulting in rapid, controlled droplet formation, and is used in applications like inkjet printing. Dripping involves a more passive process where droplets form as liquid drips into another fluid, suitable for simple droplet generation. Threading displaces one fluid with another, creating continuous threads without droplet formation, commonly employed for controlled mixing. Tubing maintains the continuous flow of one fluid inside a microchannel while another surrounds it and is useful for uninterrupted flows. Viscous displacement relies on fluid viscosity differences to displace one fluid with another and is used for precise fluid manipulation.

Jetting, dripping, threading, tubing, and viscous displacement flow patterns were observed for the flow-focusing device [234]. Figure 9A shows the droplet of the dispersed phase that breaks far from the focusing section (jetting pattern), while in Figure 9B, the droplet breaks and retracts, forming a droplet near the focusing section (dripping pattern). However, for threading, tubing, and viscous displacement patterns, the concept behind the droplet production technique is different. For the threading regime and when the droplet is stable and does not break, a droplet pattern is observed (Figure 9C). The tubing pattern (Figure 9D) has the same concept of threading, filling almost the whole cross-section of the main channel with the dispersed phase, but when the flow rate is too high, the viscous displacement regime happens, and the dispersed phase starts to fill the inlet channels of the continuous phase (Figure 9E) [47]. Therefore, analyzing the particle size is a bit challenging because of geometry dependence and interplay with regime changes. Carneiro et al. [234] used PDMS for hydrogel droplet generation and showed that the capillary number of the dispersed phase has an impact on the size of droplets where the effect is nonmonotonic and depends directly on the flow regime. Microfluidic droplet generation ensures that droplets contain a consistent number of cells and nutrients, promoting uniformity and reproducibility in experimental data. This technique also shields cells from shear stress by encapsulating them within small droplets, safeguarding them from direct exposure to the surrounding fluid. This is particularly advantageous for sensitive STEM cells that are susceptible to mechanical stress. In a study by Gwon et al. [235], a coaxial-flow-focusing device was employed to trap human pluripotent stem cells (hPSCs) within core-shell microcapsules. For hPSCs enclosed in poly(ethylene glycol) spheroids, viability exceeded 95%, while those enclosed in solid gel particles (lacking an aqueous core) exhibited less than 50% viability three days after encapsulation [235]. Orabi et al. [43] investigated the cell viability of mesenchymal stem cells (MSCs) encapsulated in 4.8 kPa and 6.7 kPa alginate–gelatin hydrogels. Results showed that, irrespective of the passaging history of MSCs, higher metabolic activity was observed with increased matrix mechanics of hybrid gels [43].

### 6.2. Droplet Manipulation

Fabrication and encapsulation processes using microfluidic techniques enable the generation of monodispersed hydrogel droplets where some procedures such as droplet fission, droplet fusion, and droplet sorting may be required [228]. The scale-up droplet formation phenomena can be applied by sequential droplet fission, which can be attained via active or passive methods [228]. The difference between the two methods is that the passive method uses shear forces created by the designs for the splitting of droplets; however, active methods use external power or electrical forces to split droplets [222,223,228]. Song et al. showed complete fission of dextran-rich sub-droplets after total decomposition of the fibril networks in the PEG-rich continuous phase of the droplets [236]. For droplet fusion, the droplets can be merged together, creating droplets with a larger diameter, which enables particle reaction control [222,228]. Wang et al. [237] reported that 3D-reconstructed mesovasculature models are used to guide the printing of cell droplets alongside extruded support materials such as agarose. After droplet fusion and removal of the agarose support, a perfusable branching mesovasculature is fabricated [237]. Droplet sorting is a technique used in the study of isolated droplets, purification of droplets, and control of polydispersed droplet mixtures [223,230]. This technique can be enhanced via channel geometry based on the size of droplets or gravitational force or even electrical forces [222,228]. Droplet manipulation methodologies afford precise regulation over microenvironmental parameters, thereby engendering the capacity for high-capacity screening initiatives, expediting the facilitation of single-cell analytical pursuits, orchestrating spatial arrangements, effectuating the establishment of concentration gradients, facilitating time-resolved investigative endeavors, and delving into intercellular interplay. Consequently, these attributes collectively establish a versatile substrate amenable to the comprehensive investigation of stem cell co-culture dynamics and differentiation phenomena. As a result, the elucidation of these multifaceted aspects significantly advances the comprehension of stem cell comportment and the intricacies of differentiation processes, thereby conferring notable contributions to the realms of regenerative therapeutics, pathological emulation, and pharmaceutical exploration.

An innovative approach with the potential to revolutionize intra/extravasation studies in microfluidics involves the application of droplet manipulation techniques. The utilization of circulating tumor cells (CTCs) as biomarkers in liquid biopsy holds promise in addressing this challenge because of their pivotal role in cancer metastasis, encompassing intravasation, circulation, extravasation, and secondary tumor formation. The advancement of microfluidics permits in vitro modeling of cancer metastasis through microenvironment mimicking, enabling comprehensive analysis and monitoring. The imperative understanding of cancer cell extravasation and metastatic colony formation underscores its significance in cancer research. Droplet manipulation recreates conditions for cancer cells to traverse vessel walls and establish secondary tumors, allowing investigation of molecular and physical determinants. Moreover, it facilitates the creation of microenvironments supporting the interaction and migration of diverse cell types, shedding light on cellular dynamics during immune response and inflammation in extravasation.

### 6.3. Surfactants

One of the major problems scientists and researchers are facing in hydrogel droplet generation is coalescence. Coalescence is defined as the merging of two droplets into one and usually happens before the gelation of hydrogel droplets. Surfactants are added into one of the phases in dispersed or continuous channels to prevent coalescence and stabilize the droplet interface [47]. Surfactants are amphiphilic molecules that alter the interface properties because of the two different functional groups they have, which are the hydrophilic and oleophilic groups [221]. The physical reason behind adding surfactants is to coat the interface area to reduce the interfacial tension below the value at the critical micelle concentration (CMC) [221,238]. Surfactants change the rheological properties of the interface, mainly the viscosity and elasticity, which directly impact the particle size [239].

Mass transport limitations directly impact the distribution of surfactants in the flow, which obstructs droplet formation and stability [47]. When the adsorption time of the surfactant into the interface is longer than the formation time of a new droplet, coalescence happens [221]. Two major effects that are observed in microfluidic droplet generation are the Marangoni effect and Ostwald ripening effect [47]. Marangoni effects are defined as a non-uniform distribution of the surfactants at the surface of the droplet, which creates a concentration gradient that generates a stress opposed to the flow direction and rigidifies the interface [221]. Marangoni forces directly impact the flow around the interface and influence the droplet size, which prevents coalescence. The creation of large droplets caused by the coalescence of small ones is known as the Ostwald ripening effect [47]. The physical definition behind this effect is that the high Laplace pressure in small droplets leads to dissolution and merging into the bigger ones [221]. The hydrophobic molecules or surfactants are added to avoid both effects [240]. Span 80 is widely used in hydrogel droplet production in microfluidics and is largely used in the pharmaceutical industry because of its properties and effects in emulsions at low concentrations [221,241]. Span 80 is known as a non-ionic surfactant, and it can be used with different hydrocarbon oils [221]. The concentration of surfactants such as span 80 has shown a direct impact on the size of droplets, interfacial tension, and capillary numbers [47]. When the concentration of surfactants increases, the interfacial tension decreases, leading to high capillary numbers and smaller droplet size [240]. As mentioned earlier, PDMS is the most widely used biomaterial for channel surfaces in droplet microfluidic generation because of its highly hydrophobic nature. The hydrophobicity of PDMS completely prevents the formation of oil in water droplets even without prior surface treatment. However, in some applications such as the formation of vesicles and the encapsulation of single cells in double emulsions, it is necessary to generate oil in water droplets. Different techniques were followed for the hydrophilicity enhancement of PDMS including UV or plasma irradiations, a coating with a hydrophilic polymer, or the incorporation of amphiphilic surfactant in the PDMS bulk. Ho et al. [242] reported that the encapsulation of cell-free expression (CFE) systems derived from mammalian cells within double-emulsion templated vesicles resulted in the aggregation of actin protein in the presence of poly(vinyl) alcohol surfactant. Furthermore, surfactant-templated chitosan-based hydrogels have exhibited potential as viable substrates for both stem and neural cells. The templating approach effectively demonstrated the modulation of material attributes while preserving chemical composition, thereby evoking analogous cellular responses in comparison to non-templated counterparts [243]. Consequently, surfactants possess the capacity to induce alterations in flow shear characteristics through mechanisms encompassing surface-tension reduction, viscosity adjustment, interfacial perturbations, boundary-layer generation, emulsification, and dispersion influences, along with boundary lubrication impacts. The precise manifestations of these effects are contingent upon surfactant typology, concentration, fluidic composition, and ambient circumstances.

### 6.4. Hydrogel Rheology in Droplet Microfluidics

The rheological attributes of hydrogels exert a pronounced influence on their conduct within microfluidic frameworks. Rheology, a discipline concerned with how substances deform and flow in reaction to applied forces, encompasses traits such as viscosity, elasticity, and shear thinning. In contrast to water, hydrogels tend to exhibit elevated viscosity, particularly at higher concentrations. This augmented viscosity can impact the flow dynamics within microfluidic conduits, possibly necessitating elevated pressures or prolonged durations to transport hydrogel solutions through microchannels compared to water, which may, in turn, impact droplet size [244]. Hydrogel solutions commonly manifest shear-thinning behavior, signifying a reduction in viscosity as the shear rate (flow velocity) escalates. In microfluidic apparatuses, variations in flow velocity across diverse zones of the system can lead to dissimilar levels of shear stress. This shear-thinning phenomenon can impinge upon the flow profile and dispersion characteristics of hydrogel solutions inside microfluidic channels [245].

Hydrogel solutions may also exhibit a degree of elasticity, especially at higher concentrations. This elasticity can elicit a rebound or retraction of the fluid when subjected to an external force, a phenomenon recognized as viscoelastic behavior. Within microfluidic systems, this viscoelastic response can exert an influence on the precision and predictability of fluid motion [246]. Hydrogels are commonly employed for the encapsulation of particles or nanoparticles, and the rheological traits of these solutions can affect the suspension and transportation of these particles within microfluidic platforms. The viscosity and elasticity of hydrogel solutions may influence the spatial distribution and motion of particles within the fluid.

Notably, the rheological attributes of hydrogel solutions have the capacity to influence interactions with biological molecules, cells, and tissues within the microfluidic milieu. This influence extends to processes such as cell encapsulation, drug delivery, and tissue engineering. Consequently, hydrogel solutions and water are characterized by contrasting rheological properties, with hydrogels typically demonstrating elevated viscosity, potential shear-thinning behavior, and viscoelasticity. These distinctions can wield a substantial impact on the behavior and efficacy of hydrogels in microfluidic systems, substantially impacting droplet size. Therefore, it is crucial to consider the unique rheological properties of hydrogels when designing and using microfluidic devices for applications involving these materials. Additionally, the depiction of viscoelasticity in physiological tissues involves a blend of experimental assessments, mathematical modeling, and imaging methods. This characterization plays a pivotal role in comprehending the mechanical properties of tissues, devising medical equipment, and formulating treatment approaches for diverse health conditions.

## 7. Droplets Shearing and Potential Impact on Cell Physiology and Differentiation

In the last decade, the effect of shear stress on cell behavior has been widely studied, where different cell lines such as mesenchymal stem cells, adipocytes, endothelial cells, and beta cells were used [240]. Shear stress has been found to affect the binding and uptake of extracellular cargo in which the cells react to shear stress by changes in ion channel activation, gene expression, and reorganization of the whole cell layer [136]. Applying shear stress on cells in microfluidic devices has been implemented by many researchers. However, to our knowledge, no one has yet investigated the impact of shear stress on cell-laden hydrogel droplets, which is most applicable to future hydrogel-based bioprinting and tissue regeneration. For example, Yang et al. studied the effect of shear stress on human adipose-derived stem cell (ADSC) differentiation into adipocytes using microfluidics [247]. There, a decrease in adiponectin secretion and an increase in free fatty acid secretion were reported while increasing shear stress [247]. Siddique et al. coated the PDMS surface of a microfluidic device with collagen using 3-aminopropyl triethoxysilane (APTES) and studied the cell adhesion under shear stress [248]. In microfluidic devices with APTES-anchored collagen, cells showed better adhesion and proliferation at shear stresses between 11.6 and 93 dyn/cm^2^ [248]. Yu et al. investigated the effects of low fluid-induced stresses on osteoblasts using a microfluidic-based multi-shear device [249]. MC3T3-E1 cells were promoted to proliferate and differentiate with fluid-flow-induced stress (FSS) ranging from 1.5 to 52.6 µPa; however, FSS over 412 µPa inhibited the proliferation and differentiation of cells [249]. Zhao et al. studied the effects of orbital shear stress on human mesenchymal stem cell proliferation, morphology change, osteogenic differentiation, and Notch1-D114 signaling [250]. Osteogenic differentiation was studied by characterizing alkaline phosphate (ALP) activity, and the modulation of orbital shear stress on Notch1-D114 signaling was investigated. The Notch1-D114 signaling was reported to be involved in orbital-shear-regulated osteogenic differentiation, and its inhabitation was found to mediate the effects of shear stress on human osteogenic differentiation [250].

In comparison, droplet shearing provides improved shear uniformity over traditional microfluidic shear studies. The small droplet size in droplet shear ensures a more uniform and consistent shear compared to traditional microfluidic shear studies, where the laminar flow bears a heterogeneous velocity profile [251]. Droplets also provide better control over the shear rate where the droplet immobilization and manipulation enable precise control over the shear rate, which is difficult to achieve in standard laminar microfluidic shear studies. It also offers a reduced sample consumption, which means droplet shearing requires significantly smaller sample volumes compared to traditional microfluidic shear studies, which can be particularly useful for rare or expensive samples. Therefore, droplet shearing improves accuracy, precision, and versatility compared to traditional microfluidic shear studies, making it a valuable tool for a wide range of tissue-engineering droplet-based applications.

In fundamental terms, microfluidics affords a versatile platform facilitating the manipulation of hydrogel porosity through precise control of fabrication parameters, the generation of controlled gradients, the utilization of emulsion templating, the regulation of fluid flow dynamics, and the incorporation of diverse materials. These capabilities are paramount in customizing hydrogel attributes to align with specific applications encompassing tissue engineering, pharmaceutical delivery, and the advancement of biomaterials. Microfluidics presents a pliable and intricate foundation for the co-cultivation of 3T3-L1 cells alongside endothelial cells. This technological framework encompasses refined spatial governance, the emulation of microenvironmental conditions, the simulation of fluid flow, the expeditious screening of multiple conditions, real-time observational capabilities, the generation of concentration gradients, the modeling of cellular barriers, and the facilitation of extended-term investigations. These proficiencies propel our comprehension of the reciprocal interactions between 3T3-L1 cells and endothelial cells, thereby contributing to the realm of research concerning tissue maturation, angiogenesis, and metabolic disorders. Surfactants exhibit multifaceted impacts on the modulation of shear forces within microfluidic systems. Their influences encompass the alteration of surface tension, the enhancement of flow stability, the modulation of rheological properties, the promotion of boundary lubrication, the facilitation of emulsification and mixing, the augmentation of mass transport, and the modification of interfacial characteristics. A comprehensive grasp of how surfactants govern shear forces in microfluidic contexts is indispensable for the design and optimization of microscale fluidic systems spanning diverse domains, including biological studies, chemical analyses, and materials science pursuits.

## 8. Summary

In this review, we surveyed the materials, fabrication techniques, and hydrogel selection that can best optimize microfluidic droplet generation. We identified key choices in each of these sections that would enable novel applications in shear-related tissue engineering studies. We urge our research community to explore these microfluidic and 3D printing techniques to enable novel studies of mechanotransduction in tissue engineering.

In reviewing the materials for microfluidic fabrication, we illustrated the pros and cons of silicon, glass, and rigid polymers. They were shown to be comparable with other materials because of their photochemical properties, chemical resistance, and solvent compatibility, in addition to having reliable electroosmotic flow, optical characteristics, and resistance to organic solvents. However, major drawbacks include substrate opacity that prevents fluorescence imaging, limited design freedom for complex microfluidics, and lower resolution and surface quality of rigid polymer devices. In contrast, PDMS is transparent, has more fabrication flexibility, and incurs lower costs compared to alternative materials. PDMS is also highly permeable to gases, especially oxygen and carbon dioxide, and biocompatible, making it an ideal material for microfluidic hydrogel droplet generation and tissue engineering.

Next, we reviewed how 3D printing has enabled the fabrication of complex microfluidics. Three-dimensional printers are used extensively in microfluidic applications, and fused deposition modeling has many advantages as a reliable technique for device fabrication, especially for cell-sensitive applications such as droplet encapsulation. Acrylonitrile butadiene styrene (ABS) and polylactic acid (PLA) filaments are the two most widely used materials in FDM 3D printing. PDMS can be easily integrated with the FDM printing of microfluidic devices where, after finishing printing, the prepolymer is poured and left to completely crosslink. Three-dimensional-printed microfluidic devices have grabbed the attention of researchers working with hydrogel droplets, who found that alginate, chitosan, GelMA, and hyaluronic acid have a good impact on forming cell-friendly droplets. We argue that the combination of FDM 3D printing and PDMS molding leverages the best aspects of simple 3D fabrication with well-established biosensing and biocompatibility to help accelerate tissue engineering. Using this novel approach, we can envision the creation of GelMA microtissues for studying diabetes, hyaluronic acid and alginate for cartilage regeneration, and chitosan for 3D breast cancer tissues, among others.

We reviewed the hydrogel materials used in microfluidic droplet generation and discussed their suitability for cell-based applications. Among the available hydrogels, alginate, chitosan, GelMA, and hyaluronic acid have shown great potential to generate cell-laden droplets. Specifically, GelMA presents an overall choice for enhancing the mechanical stability of gels, as well as the ability to generate shape-controlled microgels that encourage cell-responsive behavior. Furthermore, the physics of hydrogel droplet generation was reviewed. Microfluidics provides precise control over the size, shape, matrix structures, and mechanical properties of hydrogel droplets. Moreover, studies have shown the importance of surfactants in controlling these hydrogel properties, as well as downstream droplet manipulation in subsequent microchannels. In our review, we saw that the flow regime/pattern and droplet sizes are directly dependent on the rheological properties of the fluids in microchannels. To avoid coalescence in hydrogel droplets, surfactants must be chosen upon the physical, mechanical, and chemical properties of the biomaterials used to generate the hydrogel droplets. This highlights the interdependence of substrate material, fabrication technique, hydrogel, and microfluidic techniques that make droplet generation complex.

Droplet microfluidics has shown exciting prospects for various applications, and hydrogel-based droplet microfluidics, in particular, offers unique advantages. However, it also comes with challenges and research gaps that need to be addressed for its continued advancement. Hydrogel-based droplet microfluidics holds great promise in biomedical applications such as drug delivery, tissue engineering, and regenerative medicine. It allows for precise control over the encapsulation and manipulation of cells and biomolecules within hydrogel droplets. This technology enables high-throughput drug screening, which can lead to the discovery of new drugs and therapies. Hydrogel droplet microfluidics can also be used for 3D bioprinting, enabling the fabrication of complex tissue structures with high precision and cell viability. Researchers can use hydrogel droplets to compartmentalize and study biological reactions, making them valuable for synthetic biology applications. However, controlling the gelation process within droplets can be challenging, and achieving uniform crosslinking and gelation across all droplets is essential for consistent results. Maintaining droplet stability over extended periods is crucial, especially for long-term cell culture experiments. Preventing droplet coalescence or droplet evaporation is a challenge. Developing efficient methods for sorting and analyzing individual hydrogel droplets is essential for downstream applications. This includes techniques for extracting contents from droplets or analyzing encapsulated cells.

The long-term viability of cells encapsulated in hydrogel droplets still requires extensive research. Understanding and optimizing the conditions for sustained cell growth and function within droplets is a research gap. Exploring the encapsulation of multiple materials, such as different types of hydrogels or nanoparticles, within a single droplet or the generation of multimodal droplets (e.g., combining hydrogels with magnetic particles) presents exciting opportunities but also requires further investigation. Bridging the gap between laboratory research and clinical applications is a significant challenge, and demonstrating the safety and efficacy of hydrogel-based droplet microfluidics in clinical settings is an area of ongoing research.

## Figures and Tables

**Table 1:** A comprehensive analysis of the advantages and disadvantages of silicon, glass, and polymeric materials in microfluidics.

	Advantages	Disadvantages	References
Silicon	Surface modification and chemical properties	High elastic modulus	[63,65,66,67,68,69]
	Process compatibility	Lack of optical transparency	
	Resistivity and temperature relationship	Weak electrostatic forces	
	Thermoconductivity	Limited adoption	
	Electroosmotic mobility		
Glass	Optical transparency	Harsh fabrication techniques	[48,70,71,72,73,74,75,76,77,78,79,80,81,82,83,84]
	Low fluorescence background	High bonding temperatures	
	Surface stability	Limited design freedom	
	Chemical resistance	Limited integration techniques	
	Biological compatibility	Incompatibility with hybrid components	
	Electrical insulation	High material cost	
	High transmittance		
	Processing accuracy		
	Hydrophilic surface		
	Mass productivity		
Polymeric Materials	Versatile material	Inherent variability	[60,85,86,87,88,89,90,91,92,93,94,95,96,97,98,99,100,101]
	Optical transparency	Cleanroom requirements	
	Prototyping	UV opacity of PDMS	
	Mass production	UV transparency variability	
	Biocompatibility	Complex fabrication	
	Low toxicity	Limited integration techniques	
	Gas permeability	Cost of glass-based PDMS devices	
	Easy bonding	Limited oxygen permeability control	
	Flexible molecular structure		

**Table 2:** Three-dimensional printing technology used in droplet microfluidics for labs (custom) and industry (model).

Printing Technology	Manufacturer	Material	Resolution (x, y) µm	Ref
Stereolithography (SLA)	Custom	Custom resin	18 × 20	[140]
	Model	Acrylate-based resin	56 × 56	[141]
Multi-Jet Modeling (MJM)	Custom	Full Cure 720 resin	375 × 375	[124]
	Model	ABS	68 × 68	[141]
Fused Deposition Modeling (FDM)	Custom	ABS	100 × 50	[142]
	Model	ABS	100 × 100	[141]

**Table 3:** A thorough comparison of the advantages and disadvantages of the methods used to create 3D-printed microfluidic devices.

	Advantages	Disadvantages	Applications
Stereolithography (SLA)	High-precision 3D printing	Stress fractures and roughness	Immunomagnetic separation of bacteria [109], cell separation with helical channels [110], gradient generation [111], emulsion droplet generators [111,112], DNA assembly [113], microfluidic droplet generator [115], 3D parallelized microfluidic droplet generator [116]
	Digital sectioning	Vat depth limitation	
	Constrained-surface approach	Oxygen inhibition	
	Faster curing	Complex separation setup	
	Continuous printing	Material viscosity	
	Microchannel fabrication	Resolution considerations	
	Customizable droplet sizes		
	Parallelized microfluidic devices		
Multi-jet Modeling (MJM)	Multi-material printing capability	Limited temperature resistance	Anatomically accurate models for medical surgeries [118,119,120], microfluidic applications [102], pharmacokinetic profiling of drugs [121,122], microfluidic mixer and homogenizer [124], sodium alginate microspheres [125]
	High resolution	Sacrificial material requirement	
	Complex shapes and structures	Limited support material removal	
	Biocompatibility studies	Expensive technique	
		Limited in microfluidics	
Fused Deposition Modeling (FDM)	Low cost and simplicity	Poor resolution and surface finish	Interconnects [129], electrodes within biological tissue [130], microchannels in microfluidics [131], 3D-printed nervous system [132], biomimetic scaffold assembly [132], 3D microfluidic molds [102], endothelial-cell-lined vascular network [133], microfluidic channel network [134], droplet microfluidics [137,138]
	High reliability	Weak seals between layers	
	Customization	Nozzle and filament size limitations	
	Crosslinking for intra-layer strength	Resolution limitation in droplet microfluidics	
	Versatile material compatibility	Dimensional deviations	
	Biological tissue integration		
	Assembly of biomimetic scaffolds		
	Creation of microfluidic molds		
	Versatility in microfluidic applications		

**Table 4:** Comparison between advantages and disadvantages of biomaterials used for hydrogel droplet generation.

	Advantages	Disadvantages	References
Alginate	Biocompatibility and biodegradability	Shear-induced damage with high viscosities	[143,149,156]
	Low cost	Limited shape fidelity for 3D printing	
	Mild gelation abilities	Magnetic templating challenges	
	Wide structural similarity	Limited integration of bioactive molecules	
	Versatile applications	Complex composite hydrogels	
	Control over physical properties		
Chitosan	Biocompatibility	Source dependency	[157,158,159,160,161,162,163,164,165,166,167,168,169,170,171,172,173,174,175]
	Biodegradability	Limited mechanical strength	
	Osteoconduction	Potential allergenicity	
	Porous structure	Processing challenges	
	Ease of modification and processing	Limited temperature resistance	
	Precise microfluidic fabrication	Biocompatibility variation	
	Multifunctionality	Regulatory considerations	
	Membrane applications	Hydrophobic drug compatibility	
	Drug delivery		
GelMA	Biocompatibility	Hydrophilic nature	[176,177,178,179,180,181,182,183,184,185,186,187,188,189,190,191,192,193,194]
	Tunable mechanical properties	Material concentration	
	Porous scaffold formation	Limited temperature resistance	
	Precise microfibers	Regulatory considerations	
	Microfluidic fabrication	Microdroplet generation challenge	
	Gradient pore sizes		
	Enhanced mechanical strength		
	Versatile bioink		
Hyaluronic acid	Biocompatibility	Complex properties	[195,196,197,198,199,200,201,202,203,204,205,206,207,208,209,210,211]
	Water-holding capacity	Aggregation with proteoglycans	
	Tissue repair		
	Therapeutic potential		
	Easy scale-up		

**Table 5:** Size of droplets generated from microfluidic devices fabricated using stereolithography, multi-jet modeling, and fused deposition modeling techniques.

Biomaterials	3D Fabrication Techniques	Droplet Size (µm)	Ref
Alginate	Stereolithography (SLA)	70–115	[212]
	Multi-Jet Modeling (MJM)	200	[124]
	Fused Deposition Modeling (FDM)	100	[213]
Chitosan	Stereolithography (SLA)	378.2	[161]
	Multi-Jet Modeling (MJM)	<50	[214]
	Fused Deposition Modeling (FDM)	-	-
GelMA	Stereolithography (SLA)	200	[215]
	Multi-Jet Modeling (MJM)	-	-
	Fused Deposition Modeling (FDM)	-	-
Hyaluronic Acid	Stereolithography (SLA)	-	-
	Multi-Jet Modeling (MJM)	-	-
	Fused Deposition Modeling (FDM)	-	-

## Data Availability

Not applicable.
